# Are Survival Outcomes Different for Young and Old Patients with Oral and Oropharyngeal Squamous Cell Carcinoma? A Systematic Review and Meta-Analysis

**DOI:** 10.3390/cancers14081886

**Published:** 2022-04-08

**Authors:** Swagatika Panda, Neeta Mohanty, Saurav Panda, Lora Mishra, Divya Gopinath, Alkananda Sahoo, Sumanth Kumbargere Nagraj, Barbara Lapinska

**Affiliations:** 1 Department of Oral Pathology and Microbiology, Institute of Dental Sciences, Siksha ‘O’ Anusandhan Deemed to be University, Bhubaneswar 751030, India; neetamohanty@soa.ac.in (N.M.); alkanandasahoo@soa.ac.in (A.S.); 2 Department of Periodontics and Implantology, Institute of Dental Sciences, Siksha ‘O’ Anusandhan Deemed to be University, Bhubaneswar 751030, India; sauravpanda@soa.ac.in; 3 Department of Conservative Dentistry and Endodontics, Institute of Dental Sciences, Siksha ‘O’ Anusandhan Deemed to be University, Bhubaneswar 751030, India; loramishra@soa.ac.in; 4 Department of Oral Diagnostics and Surgical Sciences, School of Dentistry, International Medical University, Kuala Lumpur 57000, Malaysia; divyagopinath@imu.edu.my; 5 Saveetha Dental College, Saveetha Institute of Medical and Technical Sciences, Saveetha University, Chennai 602105, India; 6 Head of the Department, Oral Medicine and Oral Radiology, Faculty of Dentistry, Manipal University College, Melaka 75150, Malaysia; sumanth@manipal.edu.my; 7 Department of General Dentistry, Medical University of Lodz, 92-213 Lodz, Poland

**Keywords:** oral carcinoma, overall survival, disease-free survival, oropharyngeal squamous cell carcinoma, recurrence, distant metastasis, second primary, systematic review

## Abstract

**Simple Summary:**

Oral cancer was considered a disease of old age. However, there has been a recent surge in the incidence of oral cancer in young individuals. Age dependence on survival outcomes such as overall survival, disease-free survival, recurrence, distant metastasis and second primary in surgically treated oral cancer has been investigated several times and the results differ. This systematic review and meta-analysis has been conducted to address this concern. The results of the present research may facilitate age-dependent prognosis stratification, which would assist in treatment planning in oral cancer patients.

**Abstract:**

This systematic review and meta-analysis aims to address whether age can be a determinant of overall survival (OS), disease-free survival (DFS), recurrence, distant metastasis (DM) and second primary (SP) in surgically treated oral and oropharyngeal squamous cell carcinoma (OOPSCC). A total of 4981 cases and 44254 controls from 25 comparative observational studies were included in the analysis. A significantly better OS (matched subgroup analysis: OR 1.64; 95% CI 1.31–2.04, overall analysis: OR 1.48; 95% CI 1.09–2.01) was observed in young patients compared to older adults, with heterogeneity ranging from moderate to severe. Worse DFS (unmatched subgroup analysis OR 0.43; 95% CI 0.27–0.68) was observed in young patients compared to older adults with minimal to moderate heterogeneity. The frequency of recurrence (OR 1.49; 95% CI 1.10–2.02) and DM (OR 1.83; 95% CI 1.10–3.03) was significantly higher in the young patients, as found in unmatched and matched subgroup analysis, with the least heterogeneities. Young age can be considered as an independent prognostic factor for recurrence and distant metastases in OOP-SCC. Larger and methodologically robust observational studies with longer follow-up are needed to establish the definitive role of age as an independent prognostic factor on OS and DFS in OOPSCC.

## 1. Introduction

Oral and oropharyngeal cancers are the sixth-most common cancers worldwide and more than 90% of these cancers are histologically squamous cell carcinomas, termed as oropharyngeal squamous cell carcinoma (OPSCC) [1,2]. The “International Classification of Diseases and Related Health Problems (ICD-10)”, an international standard recommended by the World Health Organization (WHO currently in the 10th revision) [1,2], divides malignant neoplasms of the head and neck region into the codes C00 to C14. OSCC, according to their localizations, incorporate C01 to C6 (ICD-10), with C01: base of the tongue, C02: other and unspecified parts of the tongue, C03: alveolar mucosa and gingiva, C04: floor of the mouth, C05: hard palate, except C05.1: soft palate and C05.2: uvula, and C06: other and unspecified parts of the oral cavity including the buccal mucosa [1,2]. However, base of the tongue (C01), which belongs to oropharyngeal cancers embryologically as the posterior one-third, is developed from the third branchial arch [1,3]. OPSCC includes C01 and C10, which are driven by oncogenic variants of human papillomavirus (HPV) [1,2,4]. Smoking tobacco and alcohol consumption have been widely accepted as the major etiologic factors for OPSCC [1,2,3]. Other less common factors include betel quid chewing, a diet low in vegetables and fruits, poor nutrition, marijuana smoking, poor oral hygiene, and certain genetic mutations [1,2,3]. Clinicopathologic prognostic factors of OPSCC such as TNM staging, patient’s general health status, co-morbidities, primary tumor macrophage content, and lymph node metastasis are well studied [5,6,7,8,9]. The role of age as a prognostic factor has been proposed recently.

Literature on OOPSCC often does not follow the distinction between oral cavity and oropharynx. In fact, anatomic subsite definitions are at times vague, with some authors using the term “oral” for cancers of the oral cavity inclusive of the oropharynx [1]. Regardless, OOPSCCs are mostly referred to together [1,2,3,10], because of which we have used the same terminology. OOPSCCs are conventionally known to be a disease of the elderly population with an age predilection of more than 60 years [1,2,3,10]. Ablative surgery, with curative intent, has been the mainstay of treatment for these cancers for over a century. Surgical resection helps in accurate staging, with appropriate details on the status of margins and the spread of tumor that can help in deciding subsequent management based upon assessment of risk versus benefit. The recent shift in the demographic trend from old to young age has created controversies regarding the influence of age on the prognosis of OOPSCC after treatment [10,11,12]. Numerous early reports concluded that the disease is more aggressive, and the prognosis is poorer in young adults; hence, young adults are suggested as a distinct cohort with different risk factors and disease behavior [1,11,13]. On the contrary, certain other studies showed the absence of any difference in terms of survival in young patients [14]. Moreover, studies have also highlighted the importance of ageing in poorer prognosis and overall survival in older patients [1,2,3,10]. Therefore, we hypothesized that age can be a significant factor that can segregate the outcomes in surgically treated OOPSCC, and the objective of this systematic review and meta-analysis of observational studies is to provide a comprehensive analysis of current evidence on the impact of age on the survival outcomes commonly reported in oral oncology.

## 2. Materials and Methods

### 2.1. Data Sources and Search Strategy

**Figure 1:**
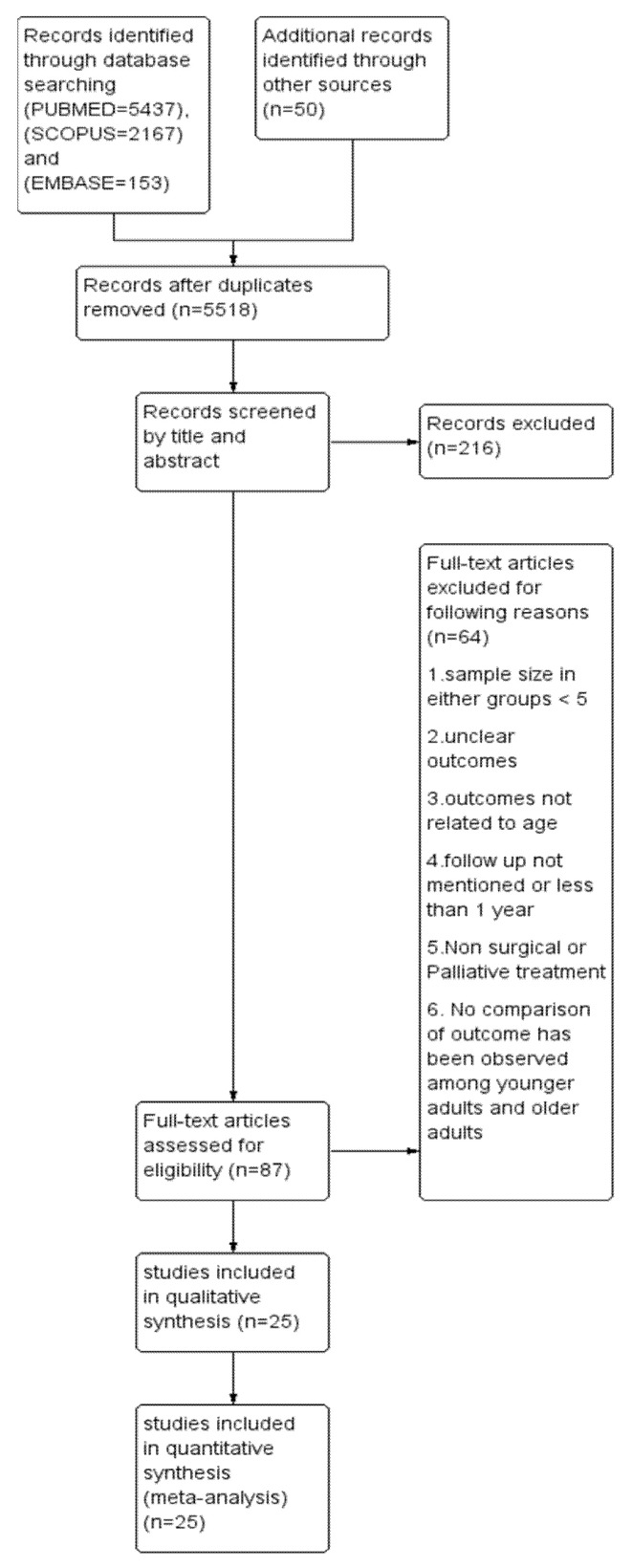
Prisma flow chart demonstrating the selection of studies.

This systematic review and meta-analysis adhered to the Primary Reporting items for Systematic review and Meta-analysis (PRISMA) guidelines (Figure 1). A well-defined protocol was prepared and registered in the International Prospective Register of Systematic Reviews (PROSPERO) with the registration number CRD42020213023. A digitalized search was carried out in electronic databases, namely, PUBMED, SCOPUS and EMBASE using the following search string: ((((((oral cancer) OR (head and neck cancer)) OR cancer, oropharyngeal)) AND (((young adults) OR age) OR less than 40 years)) AND (((((((assessment, outcomes) OR disease-free survival) OR survival) OR recurrence) OR metastasis) OR second primary cancer) OR mean survival time)). The last search was conducted on 31 December 2021. In addition, a manual search was carried out in the recent issues of dental-related journals such as Oral Oncology, Oral Surgery, Oral Medicine, Oral Pathology and Oral Radiology, European Journal of Cancer, Journal of Oral and Maxillofacial Surgery, British Journal of Cancer, and Cancer Research. The bibliography column of relevant clinical reports, potentially eligible studies, and reviews were also screened. Inclusiveness of studies followed the PICO (population, intervention, comparison, and outcome) format of observational studies.

Population: Young adult patients (≤40 years) with OSCC or OPSCC
Intervention: Surgery with or without chemoradiotherapy
Comparison: Older adult patients (>40 years) with OSCC or OPSCC
Outcomes: Overall survival (OS), Disease-free survival (DFS), recurrence, distant metastasis (DM), and second primary (SP)

### 2.2. Study Selection

Criteria for selecting studies were based upon the age group of the target population, treatment strategies, and duration of follow-up. The target population consisted of oral cancer/oropharyngeal cancer patients in two age groups. Cases included patients aged 40 years old or younger (young patients) and controls included patients older than 40 years old (old patients). Reports on patients younger than 30 years or older than 70 years were excluded. Studies that reported the outcome of OOPSCC separately while addressing that of other sites in head and neck cancer were also included. Surgery alone, or in combination with radiotherapy and/or chemotherapy, was the treatment modality in the selected articles. Studies reporting outcome/outcomes such as three- to five-year overall survival (OS), disease-free survival (DFS), recurrence, distant metastasis (DM), and second primary (SP) were included. Studies that reported the events with a follow-up for less than one year were excluded. OS is defined as the time interval between primary treatment and death due to oral cancer or last follow-up. DFS is defined as the time interval between primary treatment and the first recurrence.

### 2.3. Data Extraction and Quality Assessment

Excel Spreadsheet (Microsoft, Redmond, WA, USA) was used to retrieve relevant information from the included studies for qualitative synthesis. Data extraction was carried out separately by three independent reviewers (S.P. (Swagatika Panda), S.P. (Saurav Panda), N.M.). The authors were reached over telephone or email to enquire about the details of missing or unclear information. Parameters like demographic characteristics, study design, sample size, clinical features such as age, gender, site, Tumor, Node, Metastasis (TNM) staging, grading, follow-up duration, treatment strategies and reported outcome/outcomes in the form of a number or percentage were recorded. Due to the non-uniform presentation of staging, we have categorized cT1, cT2, stage I and II as early and the rest as the advanced stage of presentation. Similarly, we have combined the well-differentiated tumors (Grade I) and moderately differentiated tumors (Grade II) as low grade and poorly differentiated and undifferentiated as high-grade tumors.

The risk of bias in methodological quality was assessed in Review Manager 5.3 Software (Copenhagen, Denmark). by two independent investigators (S.P. (Swagatika Panda) and S.P. (Saurav Panda)). Each study was evaluated for (1) selection bias, (2) exposure risk, (3) co-morbidity, (4) attrition bias, (5) confounding bias, and (6) immortal time bias. Two authors appraised nine points in every included study and colored ‘green’ for low risk, ‘yellow’ for unclear, and ‘red’ for high risk. The risk of bias was categorized as low when the study was showing more and equal to 60% of the ‘green’ score and high when there was 40% of either ‘yellow’ or ‘red’.

### 2.4. Data Synthesis and Analysis

A detailed qualitative analysis was carried out for all included studies. A Chi-square test was performed in SPSS (IBM SPSS Statistics for Windows, Version 23.0. Armonk, NY, USA: IBM Corp.) to compare the differences in staging and grading of OOPSCC between two age groups. A *p*-value of less than 0.05 was considered statistically significant. To conduct meta-analysis, Odd’s ratio (OR) was pooled from the number or percentages of events of OS, DFS, recurrence, DM, and SP in both the cohorts. Forest plots were constructed using Review manager 5.3 (Copenhagen, Denmark). Due to the diverse nature of interventions, the follow-up period and clinicopathologic factors heterogeneity was expected and therefore the random-effects model was chosen. The heterogeneity of the included studies was assessed using I^2^ statistics. Chi-square statistics were used to measure the variation in the effect size due to heterogeneity. Values of I^2^ greater than 50% represented significant heterogeneity. The risk of bias was mapped to express the heterogeneity among eligible studies [15]. To conduct subgroup analysis, studies were further segregated into matched and unmatched studies for one or many factors, including age, gender, site, TNM staging, and treatments provided. Publication bias was assessed using the Funnel plot in Revman.

## 3. Results

### 3.1. Characteristics of Included Studies

A total of 5247, 2167 and 153 articles were identified from three databases like PUBMED, SCOPUS and EMBASE, respectively, and duplicates were removed. After careful reading of abstracts and full texts, twenty-five articles [16,17,18,19,20,21,22,23,24,25,26,27,28,29,30,31,32,33,34,35,36,37,38,39,40] were selected for conducting the systematic review (SR), followed by a meta-analysis. A PRISMA flow chart depicting the selection of articles is shown in Figure 1. Out of 25 publications, ten [16,17,18,19,20,21,27,30,32,38] were studied in Asian populations and the rest in European, Australian and North American populations. Only two reports [18,26] were prospective in nature. A total of 4981 young patients and 44,254 old patients were studied. Male-to-female ratios in case and controls were found to be 1.4 and 1.7, respectively (*p* = 0.39). Tongue is the predominant site in eight studies [19,20,22,23,30,31,33,40], whereas ten studies [16,17,28,32,34,35,36,37,38,39] were conducted exclusively in tongue. Rahman et al. [21] did not report the specific site. The proportions of an early stage in the case and control group were found to be 43.6% and 44.4%, respectively (*p* = 0.35). Two studies [21,27] did not report the staging. Grading has been reported in all except five studies [25,26,27,33,39]. The proportion of low grades in case and control were found to be 70.5% and 71.2%, respectively (*p* = 0.62). The median follow-up period as mentioned in all studies, except one [18], was found to be 59.5 months and 57.7 months in case and control, respectively (*p* = 0.52). Clinicopathological and outcome details of included articles are listed in Table 1 and Table 2, respectively.

### 3.2. Risk of Bias

Out of 25 studies, 13 studies showed a high risk of bias, as depicted in supplemental Figure 2. Comorbidity bias was the most common bias seen in 15 out of 25 articles. Selection and attrition bias were found to be the least common.

### 3.3. Meta-Analysis

#### 3.3.1. Overall Survival

**Figure 3:**
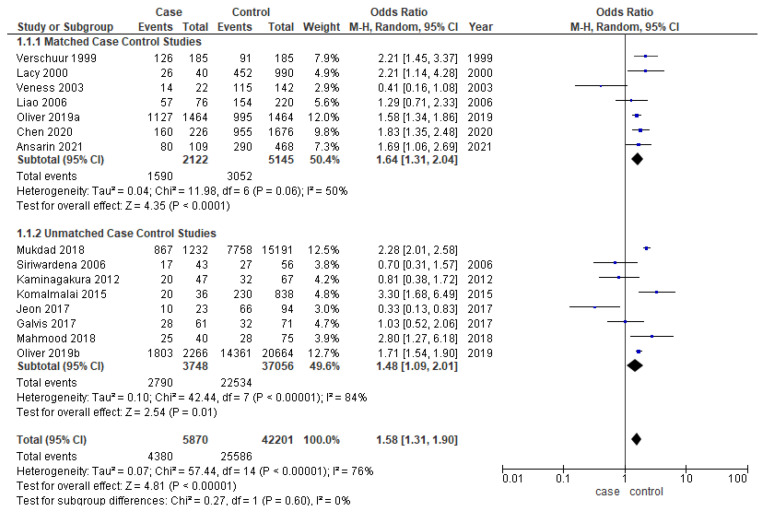
Forest plot demonstrating the OR for overall survival in young patients.

Younger patients had significantly better overall survival in the overall analysis (OR-1.58; 95% CI-1.31–1.90) (Figure 3). The cases and controls were matched for one or multiple factors such as age, gender, TNM staging, and grading in six out of thirteen studies [17,18,20,22,25,27,31,32,33,35,36,37,40]. The odds ratio for both matched (OR-1.64; 95% CI 1.31–2.04) and unmatched studies (OR-1.48; 95% CI 1.09–2.01) are similar to the overall OR. While meta-analysis of matched studies demonstrated moderate heterogeneity (50%), significant heterogeneity was identified in overall analysis (76%) and unmatched analysis (84%).

#### 3.3.2. Disease-Free Survival

**Figure 4:**
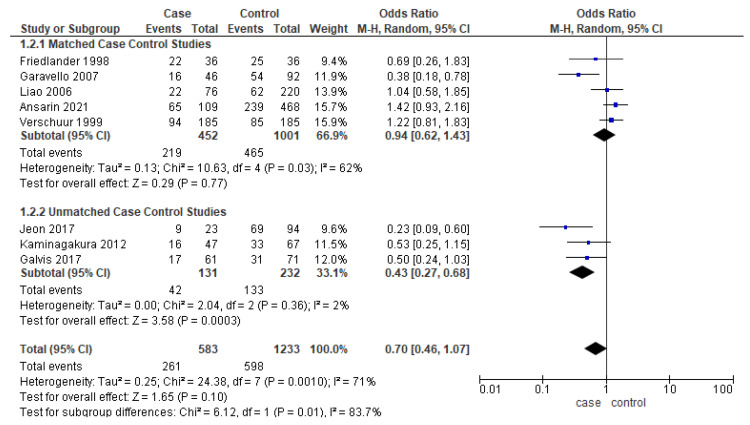
Forest plot demonstrating the OR for DFS in young patients.

Eight studies [17,22,25,28,38,39,40] reported the DFS, out of which only five studies were matched for one or more clinicopathologic factors. Unmatched subgroup analysis demonstrated worse DFS in younger adults (Figure 4). The heterogeneity remained moderate (53%) and high (70%), respectively.

#### 3.3.3. Events of Recurrence

**Figure 5:**
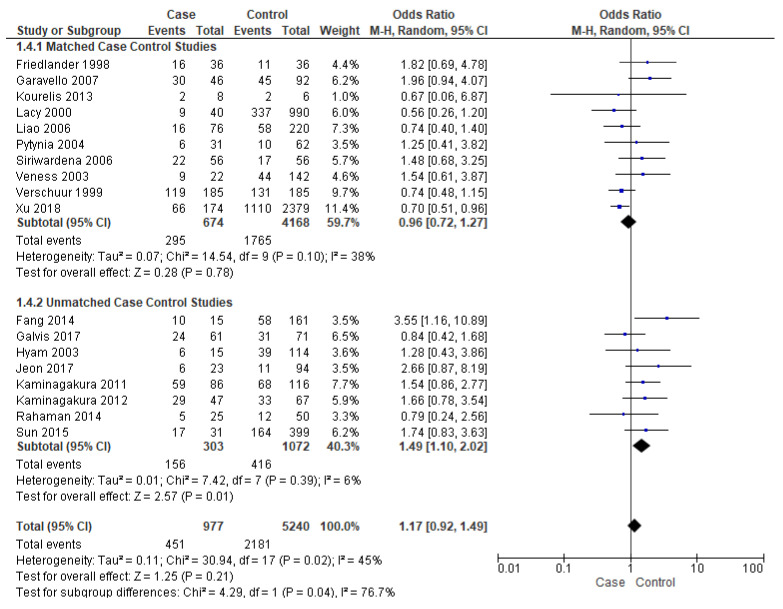
Forest plot demonstrating the OR for events of recurrence in young patients.

Events of recurrence were reported by eighteen studies [16,17,19,21,22,23,25,26,27,28,29,30,31,34,37,38,39,40]. Unmatched subgroup analysis comprised of eight studies [16,17,19,21,22,23,34,40] showed that young patients had a significantly high risk of recurrence, almost 49% (OR-1.49; 95% CI 1.10–2.02) (Figure 5), with a true population effect between 10% and 102%. The heterogeneity was very low (6%) for this comparison. However, the overall analysis and matched subgroup analysis did not find any difference.

#### 3.3.4. Distant Metastasis

**Figure 6:**
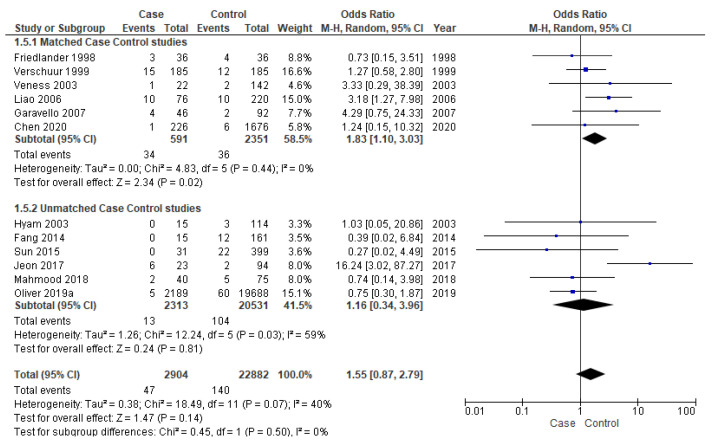
Forest plot demonstrating the OR for distant metastasis in young patients.

Twelve studies [16,17,18,19,25,28,33,34,36,37,38,39] were included for meta-analysis of DM, out of which six [25,28,33,37,38,39] were matched studies. The matched subgroup analysis illustrated a significantly higher risk (90%) of developing DM in young patients (OR-1.83; 95% CI 1.10–3.03) compared to the old ones. The heterogeneity remained nil (I^2^ = 0%) (Figure 6).

#### 3.3.5. Second Primary

**Figure 7:**
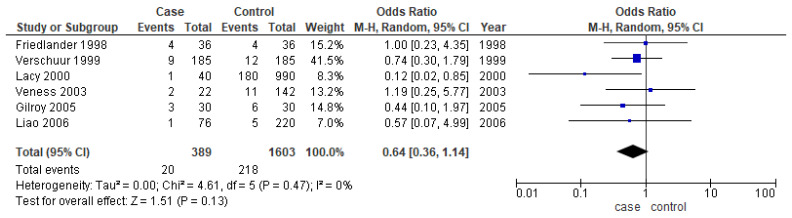
Forest plot demonstrating the OR for the second primary in young patients.

Only six studies [24,25,31,37,38,39] reported the second primary as one of their outcomes. The meta-analysis did not show any conclusive difference between the two age groups (OR-0.64; 95% CI: 0.36–1.14) (Figure 7).

#### 3.3.6. Publication Bias

**Figure A1:**
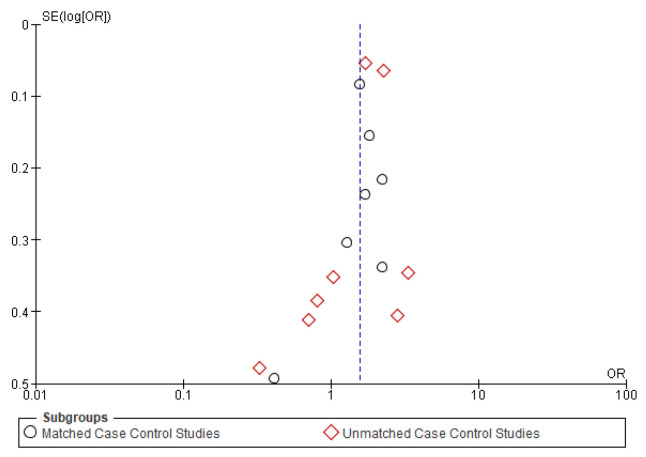
Funnel plot of overall survival.

**Figure A2:**
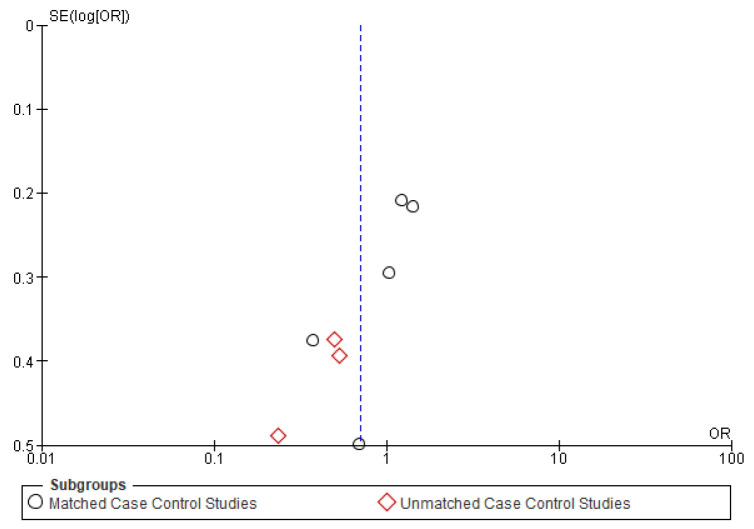
Funnel plot of disease-free survival.

**Figure A3:**
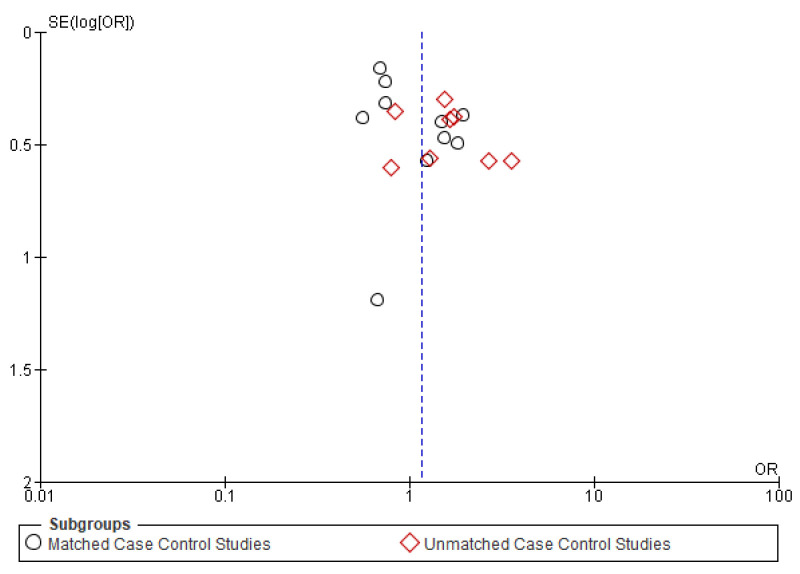
Funnel plot of recurrence.

**Figure A4:**
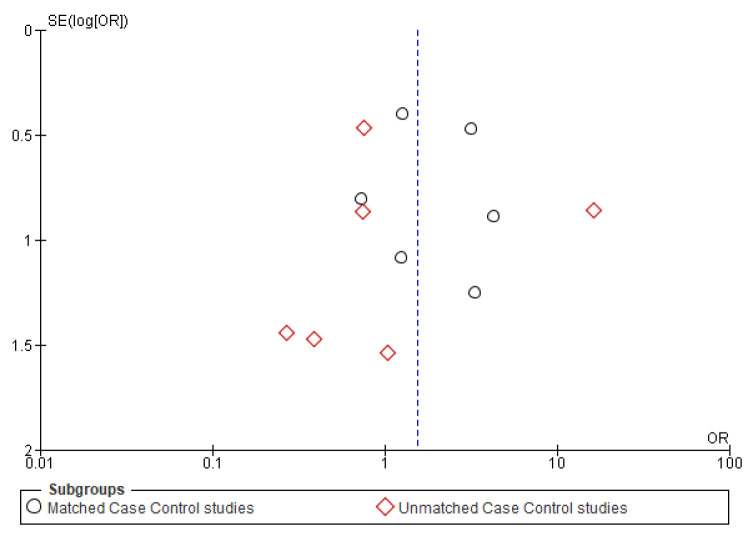
Funnel plot of distant metastasis.

**Figure A5:**
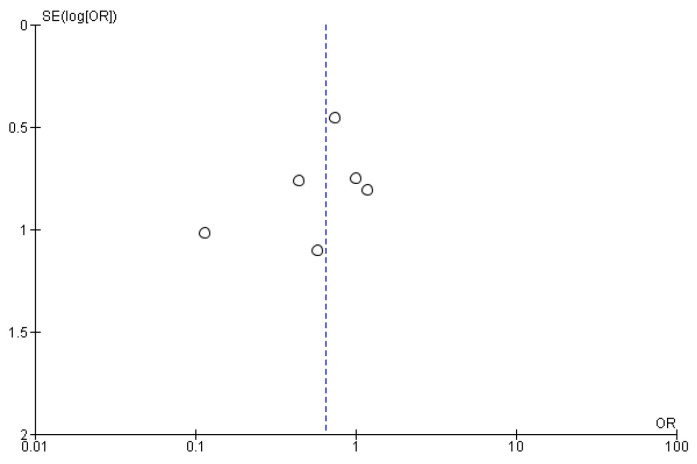
Funnel plot of second primary.

The symmetrical funnel plot of five negative outcomes (Figure A1, Figure A2, Figure A3, Figure A4 and Figure A5) indicates minimal publication bias in the matched subgroup analysis of recurrence and distant metastasis. The overall analysis of recurrence and second primary also demonstrated minimal publication bias.

## 4. Discussion

This systematic review and meta-analysis provides the cumulate evidence on age as a risk factor in stratifying negative outcomes in OOPSCC based on the observations from 25 comparative observational studies. One similar meta-analysis that compared the outcomes in older and younger adults [41] included the cutoff age for young patients as less than 30, 40, and 45 years. The present systematic review and meta-analysis has reasonably chosen less and equal to 40 years as the cutoff age of younger adults, which had been objectively identified by Marchiano et al. [42] as a transition point defining the young cohort as individuals with less and equal to 40 years. Another systematic review without meta-analysis has been reported by Sarode et al. [43], who has not determined the cutoff value of young age. The most recent meta-analysis on oral cavity squamous cell carcinoma, as reported by Lee et al. [44], suggested similar 5-year OS and DFS in both younger and older cohorts. To the best of our knowledge, this meta-analysis is the first to report a comprehensive comparison of five outcomes of OOPSCC between young and old patients. Demographic and clinicopathological features of young and old patients were also analyzed.

The present meta-analysis suggested that OS and DFS were significantly dependent upon age. Younger patients were found to present with better OS and worse DFS as compared to older adults. However, the heterogeneity incurred in the above analysis was high except for unmatched subgroup analysis for DFS (I^2^ = 2%) and matched subgroup analysis for OS (50%). The potential source of heterogeneity may be the absence of site specificity. In spite of the site-specific differences in molecular signature, most of the studies have pooled the tumors in different subsites of the oral cavity [45]. Other possible reasons for heterogeneity could be methodological issues such as patient selection (comorbidities and range of age group), type and extent of neck dissection, and large variation in the follow- up period. The significant heterogeneity could also be attributed to the differences in sample sizes, population features, and study setting. The present result does not coincide with Lee’s meta-analysis, which suggested that OS and DFS are similar in both the cohorts [44]. The difference in opinion may be attributed to Lee’s describing only oral cavity squamous cell carcinoma, in contrast to the present meta-analysis, which compared both oral cavity and oropharyngeal squamous cell carcinoma. In fact, better OS in younger adults was also observed in colorectal cancers, too. Considering the limited matched studies and heterogeneity, further research is warranted. Essentially, OS is comprised of DFS plus post-progression survival. Therefore, DFS has been suggested as a potential surrogate for OS in several cancers [46,47,48]. The correlation approach has been widely adopted to theoretically validate the efficiency of this surrogate endpoint [49]. However, better OS and worse DFS in young patients as revealed by the present result of meta-analysis may suggest that this may not prevail in OOPSCC.

This study also revealed 49% greater odds of recurrence (95% CI-1.10–2.02) in unmatched subgroup analysis and 90% greater odds of metastasis in matched subgroup analysis in young subjects. Frequent events of recurrence and DM in younger adults as evident from the results may be the reason for worse DFS compared to older patients. This inverse relationship of age with recurrence and DM can be hypothetically explained with two reasons. First, the metastatic process can be protected by the deterioration of the immune system, which happens in old age [50]. Second, the age-dependent reduction in matrix-modifying protease activity of the extracellular matrix may also prevent metastasis in old patients [51]. Age dependency of survival outcomes may be attributed to the differences at the molecular level. Few studies illustrated distinctive molecular events in the young [52,53], whereas others reported similar molecular profiles in both age groups [54,55]. Since young patients are exposed to etiological factors such as tobacco and alcohol for a shorter period, it has been speculated that risk factors other than these two could play a role in that age group [56]. A systematic review and meta-analysis recently demonstrated the role of another prognostic factor, HPV, in shortening OS and lowering DM, with it having no effect on recurrence and DFS [4]. The results indicated that young age may be considered as an independent determining factor for recurrence and DM, though more matched studies are required to reinforce the association with recurrence.

Empirical studies on follow-up on OOPSCC are scarce and most studies reported the combined data for all head and neck cancers, which encompass a group of malignancies with distinct etiology, prognosis and frequency and timing of second primaries [56,57]. The available studies have shown that SPs can adversely influence OS in older patients with OOPSCC [58,59]. However, our analysis could not generate any conclusive evidence regarding differences in the frequency of SP in the two age groups. While old age (>60 years) was found to be independently associated with frequent events of SP in head and neck carcinoma [56], another study reported that young patients (<65 years) were prone to develop SP in esophageal cancers [58]. Establishing a standardized age group classification for risk stratification is essential for conclusive evidence.

Evaluation of the demographic characteristics of the selected studies revealed that there was a paucity of studies on the Asian population, even though these countries report the highest incidence of OOPSCC in the world, which is attributed to the chewing of areca nut and smokeless tobacco, both “homemade” and commercial, and which vary considerably in composition, mode of use, and toxicity [59]. Further, a female preponderance, though non-significant, was found in our review, which is supported by a few others as well [60,61,62], whereas it is contradicted by others [11,63,64]. The staging and grading of the tumors did not show significant differences between the two groups, making both of them comparable. However, Troeltzsch et al. [65] reported that age, in general, was shown to influence staging, supported by Sasaki et al. [66] and a similar review by Pitman et al. [67]. Nevertheless, similar to our results, grading was not influenced by age in the study by Troeltzsch et al. [65], whereas our results were contradicted by Sasaki et al. [66]. So it is safer to infer that there may be the least influence of age on staging and grading in these tumors. Furthermore, the tongue was the most common subsite in the included studies and was exclusively studied in nine articles, which may have compromised the precision of this, though Bell et al. [68] reported a minimal influence of sites upon the outcome.

We present the most extensive analysis of the pooled data from twenty-three studies with subgroup analysis on matched and unmatched case-control studies along with a comprehensive comparison of both clinicopathologic and treatment-related outcomes in younger and older patients. The publication bias was minimal in the study, as illustrated by the funnel plots for all the outcomes. Nevertheless, this study has certain limitations. First, there were only a handful of case-control studies that were matched for any of the demographic parameters. Case-control studies matched based on demographic characters as well as sites, staging, grading and treatment modalities would have improved the precision of this study. Second, the information regarding the differential application of elective and therapeutic neck dissection was lacking in the included studies. Immortal time bias is not reported in any of the included studies, and this has become an unavoidable bias. Co-morbidity in either age group was not reported in ten articles, which is possibly the source of a major bias, especially while reporting OS. Inconsistency in the follow-up period in the included studies may also act as a confounding factor. While recurrence can be more frequently observed during 1 to 60 months [69], time to DM was reported to range from less than 12 months to more than 2 years [70]. Similarly, the risk of developing SP continued even for over 5 years after the diagnosis of primary oral cancer [71]. Therefore, the results of the present meta-analysis may be interpreted keeping these limitations under consideration.

## 5. Conclusions

Our results provided a comprehensive review of the differences in clinicopathologic features and survival outcomes in younger and older patients with OOPSCC. Although not sufficiently robust to recommend age-specific therapeutic and follow-up strategies, the preliminary evidence of better OS and worse DFS in young subjects mandates larger and methodologically stronger observational studies with longer follow-up periods and matched comparisons. Young age can be considered as an independent prognostic factor for assessing recurrence and DM.

## Figures and Tables

**Figure 2:**
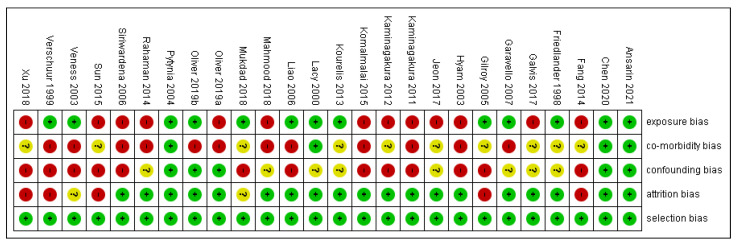
Risk of bias.

**Table 1:** Clinicopathological features of eligible articles.

Author/Year	M:F		Site		Staging						Grading					
	Case	Control	Case	Control	Case (Early)	Case (Late)	NR	Control (Early)	Control (Late)	NR	Case (High)	Case (Low)	NR	Control (High)	Control (Low)	NR
Oliver et al. (2019) [36]	1211: 1055	12,502: 8162	Tongue (E)	Tongue (E)	1225	515	526	11,529	4342	4793	1725	362	179	15,659	2956	2049
Mahmood et al. (2018) [18]	33:7	49:26	Mixed	Mixed	2	38	0	8	67	0	29	11	0	68	7	0
Galvis et al. (2018) [40]	45:16	56:15	Tongue (P)	Tongue (P)	16	45	0	14	57	0	53	36	2	62	4	5
Jeon et al. (2017) [17]	15:8	51:43	Tongue (E)	Tongue (E)	9	14	0	47	37	0	16	6	1	86	5	3
Sun et al. (2017) [17]	19:12	277:122	Tongue (P)	Tongue (P)	22	12	0	234	165	0	22	9	0	308	91	0
Komolmalai et al. (2015) [20]	23:13	494:344	Tongue (P)	Tongue (P)	14	13	0	252	411	175	29	5	2	722	62	51
Rahaman et al. (2014) [21]	NR	NR	DNA	DNA	NR	NR	0	NR	NR	0	19	6	0	37	13	0
Fang et al. (2014) [16]	6:9	113:48	Tongue (E)	Tongue (E)	12	6	0	112	49	0	10	5	0	121	40	0
Kaminagakura et al. (2011) [23]	30:17	51:16	Tongue (P)	Tongue (P)	9	38	0	17	50	0	40	7	0	65	2	0
Kaminagakura et al. (2012) [22]	65:25	99:26	Tongue (P)	Tongue (P)	14	76	0	21	104	0	63	27	0	109	4	0
Gilroy et al. (2005) [24]	NR	NR	Mixed	Mixed	3	27	0	3	27	0	19	11	0	17	13	0
Friedlander et al. (1998) [39]	20:16	NR	Tongue (E)	Tongue (E)	29	7	0	29	7	0	NR	NR	NR	NR	NR	NR
Verschuur et al. (1999) [25]	1.68:1	3.28:1	Mixed	Mixed	93	91	0	94	90	0	NR	NR	NR	NR	NR	NR
Pytynia et al. (2004) [26]	10:21:00	20:42	Mixed	Mixed	10	21	0	20	42	0	NR	NR	NR	NR	NR	NR
Siriwardena et al. (2006) [27]	4:01:00	3.7:1	Mixed	Mixed	NR	NR	0	NR	NR	0	NR	NR	NR	NR	NR	NR
Garavello et al. (2008) [28]	31:15:00	62:30	Tongue (E)	Tongue (E)	34	12	0	68	24	0	36	10	0	70	22	0
Lacy et al. (2000) [31]	29:11:00	710:280	Mixed	Mixed	18	22	0	438	552	0	36	4	0	881	109	0
Kourelis et al. (2013) [29]	NR	NR	Tongue (P)	Tongue (P)	46	23	0	46	23	0	44	25	0	44	25	0
Hyam et al. (2003) [34]	9:06:00	75:29	Tongue (E)	Tongue (E)	10	5	0	71	43	0	5	10	0	76	35	0
Mukdad et al. (2019) [35]	706:526	8895:6296	Tongue (E)	Tongue (E)	316	191	0	4215	2244	0	896	203	0	10,946	2186	0
Liao et al. (2006) [38]	71:5	194:26	Tongue (E)	Tongue (E)	37	39	0	117	103	0	73	3	0	202	18	0
Veness et al. (2003) [37]	13:9	93:49	Tongue (E)	Tongue (E)	14	8	0	91	51	0	12	6	4	110	23	7
Xu et al. (2019) [30]	109:65	1330:1049	Tongue (P)	Tongue (P)	91	52	0	1304	798	0	148	12	0	399	17	0
Ansari et al. (2021) [32]	66:43	281:187	Tongue (E)	Tongue (E)	40	69	0	184	284	0	88	19	0	365	85	0
Chen et al. (2020) [33]	135:91	1156:520	Tongue (P)	Tongue (P)	117	93	16	847	730	99	NR	NR	NR	NR	NR	NR
Total					2181	1417	542	19,761	10,300	5067	3363	777	188	30,347	5717	2115

Legend: NR = Not reported; E = Exclusive site; P = predominant site.

**Table 2:** Details of follow up, treatment and outcomes of eligible studies.

Authors	Follow Up (Months)		Number of Events									
			3–5 Year Overall Survival		Disease-Free Survival		Recurrence		Distant Metastasis		Second Primary	
	Case	Control	Case	Control	Case	Control	Case	Control	Case	Control	Case	Control
Oliver et al., 2019 [36]	6	45	1127	995					5	60		
Oliver et al., 2019 [36]	45	45										
Mahmood et al., 2018 [18]	50	55	25	28					2	5		
Galvis et al., 2018 [40]	60	60	28	32	17	31	24	31				
Jeon et al., 2017 [17]	20	20	10	66	9	69	6	11	6	2		
Sun et al., 2015 [19]	96	96					17	164	0	22		
Komolmalai et al., 2015 [20]	60	60	20	230								
Rahaman et al., 2014 [21]	60	60					5	12				
Fang et al., 2014 [16]	38.7	37.9					10	58	0	12		
Kaminagakura et al., 2012 [22]	22.2	22.2	20	32	16	33	29	33				
Kaminagakura et al., 2011 [23]	21.4	21.4					59	68				
Gilroy et al., 2005 [24]	80.4	80.4									3	6
Friedlander et al., 1998 [39]	25	51			22	25	16	11	3	4	4	4
Verschuur et al., 1999 [25]	60	60			19	51	119	131	15	12	9	12
Pytynia et al., 2004 [26]	26.5	19					6	10				
Siriwardena et al., 2006 [27]	36	36	17	27			22	17				
Garavello et al., 2008 [28]	60	60			16	54	30	45	4	2		
Lacy et al., 2000 [31]	60	60	26	452			9	337			1	180
Kourelis et al., 2013 [29]	60	60					2	2				
Hyam et al., 2003 [34]	43	43					6	39	0	3		
Mukdad et al., 2019 [35]		60										
Liao et al., 2006 [38]	28	28			22	62	16	58	10	10		
Veness et al., 2003 [37]	60	60	14	115			9	44	1	2	2	11
Xu et al., 2019 [30]	59	59					66	1110				
Ansari et al., 2021 [32]	50.6	31	80	290	65	239						
Chen et al., 2020 [33]	60	60	160	955					1	6
